# P-1429. Racial Disparities in Childhood Vaccination Rates and Their Association with State-Level Structural Racism

**DOI:** 10.1093/ofid/ofae631.1604

**Published:** 2025-01-29

**Authors:** Jin-Young Han, Yiyuan Wu, Erika Abramson

**Affiliations:** NYP/Weill Cornell Medicine, New York, New York; Weill Cornell Medicine, New York, New York; Weill Cornell Medicine, New York, New York

## Abstract

**Background:**

Racial disparities in childhood vaccination rates are well-documented nationally. These disparities may vary across states due to multiple reasons, including vaccine exemptions. A structural racism score is a measure developed to quantify systemic racial disparities across various domains, including segregation, economic status, and incarceration. Notably, structural racism is more pronounced in Northeastern and Midwestern states of the United States. Leveraging recently published Black structural racism scores at the state level, we examined the relationship between structural racism and childhood vaccination rates among non-Hispanic Black and non-Hispanic White children.

**Figure:**
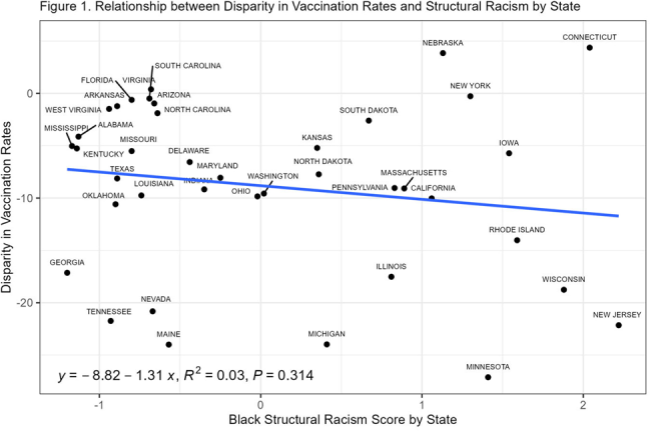

**Methods:**

We analyzed 5-year aggregate vaccination rates (2017-2022) for children aged 19-35 months using data from the US Centers for Disease Control and Prevention National Immunization Survey-Child (NIS-Child). The main outcome was the difference in percentage points between non-Hispanic Black and non-Hispanic White childhood vaccination rates for each state. The childhood vaccination rate was defined by whether children received all recommended vaccines (4+ DTaP/DTP/DT; 3+ polio; 1+ measles-containing vaccine; full series Hib; 3+ Hep B; 1+ varicella at or after 12 months of age; and 4+ PCV). Eleven states were excluded from analysis due to fewer than 30 non-Hispanic Black respondents. We investigated the relationship between state-level structural racism scores for non-Hispanic Black individuals and calculated vaccination rate differences by linear regression analyses. Additionally, we examined the relationship in states that allowed religious exemptions for childhood vaccinations.

**Figure:**
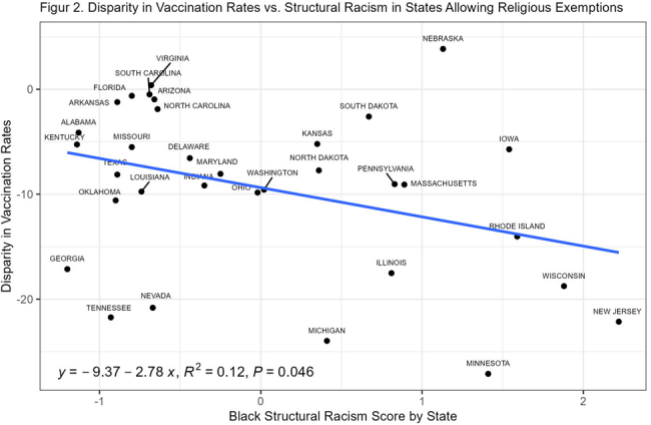

**Results:**

At the state level, higher structural racism scores were associated with greater disparities in childhood vaccination rates between non-Hispanic Black and non-Hispanic White children (Figure 1). Importantly, these associations were significant only in states that allowed religious exemptions for childhood vaccinations (Figure 2, p = 0.046).

**Conclusion:**

When childhood vaccination exemptions are permitted, state-level structural racism may significantly contribute to the observed racial disparities in vaccination rates among non-Hispanic Black children.

**Disclosures:**

**All Authors**: No reported disclosures

